# A mixed methods study protocol to identify research priorities for perioperative medicine in Australia

**DOI:** 10.1016/j.bjao.2023.100235

**Published:** 2023-10-25

**Authors:** Sophie K.A. Wallace, Tracey K. Bucknall, Andrew Forbes, Paul S. Myles

**Affiliations:** 1 Department of Anaesthesiology and Perioperative Medicine, Alfred Hospital, Melbourne, Victoria, Australia; 2 Department of Anaesthesiology and Perioperative Medicine, Central Clinical School, Monash University, Melbourne, Victoria, Australia; 3 Centre for Quality and Patient Safety- Alfred Health Partnership, Institute for Health Transformation, Deakin University, Geelong, Australia; 4 School of Nursing and Midwifery, Deakin University, Geelong, Australia; 5 Biostatistics Unit, Department of Epidemiology and Perioperative Medicine, Monash University, Melbourne, Australia

**Keywords:** anaesthesia, consumer engagement, perioperative medicine, public and patient involvement, research priorities, surgery

## Abstract

**Background:**

Clinical research in perioperative medicine requires the perspectives of patients and caregivers to increase its relevance and quality, benefiting both researchers and the community. Identifying these priorities will enable researchers, funders, and governing bodies to efficiently use scarce funding and resources. We aim to identify the top 10 research priorities in perioperative medical research in Australia.

**Methods:**

A mixed-methods, exploratory-sequential design will be conducted. The study will include five phases. Initially, a published open-ended survey gathered responses from the population (researchers, healthcare workers, and consumers) regarding uncertainties/questions relevant to the population about perioperative medical research. We collected 544 questions and quantitatively analysed and grouped them according to the Standardised Endpoints in Perioperative Medicine–Core Outcomes Measures in Perioperative and Anaesthetic Care (StEP–COMPAC) endpoints. Using multicriteria decision-making software, workshops combining the population will be conducted to determine the top 10 priorities for perioperative medicine research for the Australian population.

**Ethics and dissemination:**

Ethical approval to conduct the study was obtained from the Alfred Health (Australia) Human Research Ethics Committee (ID: 171/19). The findings will be disseminated in peer review publications, conferences, and dissemination across perioperative research networks. The top 10 priorities will be available to inform research funders, grant submissions, guidelines, and the population.

Advances in healthcare and medical treatments have enabled people to stay healthier for longer, leading to longer life expectancy and quality of life; medical research has been an essential component of this progress.^1^ Through the years, medical research has, for the most part, been ‘top-down’^2^—research conducted with little or no consultation or input from the consumer, but conceived, conducted, and published by physicians, academics, and researchers.^3^, ^4^ The populations in which the research was conducted were mostly not asked for their needs, beliefs or wants.^5^ Chalmers and colleagues^6^, ^7^, ^8^ posit that without including the consumer in the design, conduct, outcomes, and dissemination of the results, the research may not have been relevant, conclusive, and appropriate, a contributing factor to the argument that 85% of this research could be deemed as ‘waste’.^9^ It is believed that incorporating consumer engagement into contemporary research could be the ‘holy grail’ of healthcare or the next ‘blockbuster drug of the century’.^10^

The global health and medical research enterprise is of massive proportions, with budgets in the billions of dollars.^11^, ^12^, ^13^ In Australia, the National Health and Medical Research Council (NHMRC) is a Government-funded body responsible for health and medical research and the dissemination of funds.^14^ In 2019–20, the NHMRC administered 909.9 million Australian dollars.^15^ Each year, the number of submitted grants to NHMRC is increasing, with only a proportion (∼15%) successful in funding.^16^ This is identified throughout the world; the Medical Research Council (MRC) in the UK in 2021 administered 14.5^13^ million British pounds with a grant success of 21% and the National Institute of Health (NIH) in the USA administered 32.3 billion American dollars with a grant success of 19.1%.^17^

Compelling information suggests that incorporating the perspectives of the patients and caregivers in research increases the relevance and quality of the research.^3^^,^^18^ There are known disparities between the research community and the consumers of this research. Thus, collaboration is required to identify the needs and concerns of the community.^19^^,^^20^ Shaping of the research agenda for perioperative medicine research is through funders and research bodies, with little to no input from consumers being reported.^21^, ^22^, ^23^ A priority setting agenda in perioperative medicine in Australia should benefit all clinicians and guide future research in the areas of greatest need. Identifying the essential unanswered questions should lead to the reduction in ‘waste’ or ‘wrong answers’ in future research.^7^^,^^24^

One approach that has gained strong praise since its inception in 2004 is the National Institute for Health Research (NIHR)-funded James Lind Alliance (JLA),^25^ a not-for-profit initiative in the UK. The JLA brings patients, carers, and clinicians together in Priority Setting Partnerships (PSPs) to identify and prioritise the top 10 uncertainties, or unanswered questions, about the effects of treatment in various disciplines. PSPs for perioperative medicine have been conducted in the UK^26^ and Canada,^27^ but thematic analysis identified only a 50% agreement on the resultant priorities.^27^^,^^28^ The misalignment and disparity across the two continents confirm the need for a specific Australian perioperative medicine priority setting agenda.

Australia had a population of 25.7 million on 30 June 2021.^29^ A large proportion of the population, 50% was either born overseas or had parents born overseas.^30^ Australia is known as a vibrant and multicultural country with >270 ancestries. It is home to the world's oldest continuous indigenous cultures. Seven million people have migrated to Australia since 1945.^31^

Priorities identified in this project will cover all aspects of care before, during, and after anaesthesia, surgery and throughout the extended perioperative period, including the management of longer-term health problems that originate during this period.

For clarification in this process, the term ‘anaesthesia’ refers to the practice of administering medications to block the feeling of pain and other sensations, or producing a deep state of unconsciousness that eliminates all sensations, allowing medical and surgical procedures to be undertaken without causing undue distress or discomfort. The reference of consumer for the purposes of this paper refers to patients or carers of individuals having undergone surgery with anaesthesia.

Perioperative medicine refers to the healthcare received from the period from the decision to operate, which may be before hospital admission to hospital discharge and beyond.^32^^,^^33^ This does not include the actual surgery itself. The focus is on the patient's management, health, and physical well-being throughout the perioperative period and longer-term recovery.

The objective of this project is to identify the top 10 research priorities in perioperative medicine in Australia via a PSP based on the JLA methodology. The study has five phases (Fig. 1).

**Fig 1:**
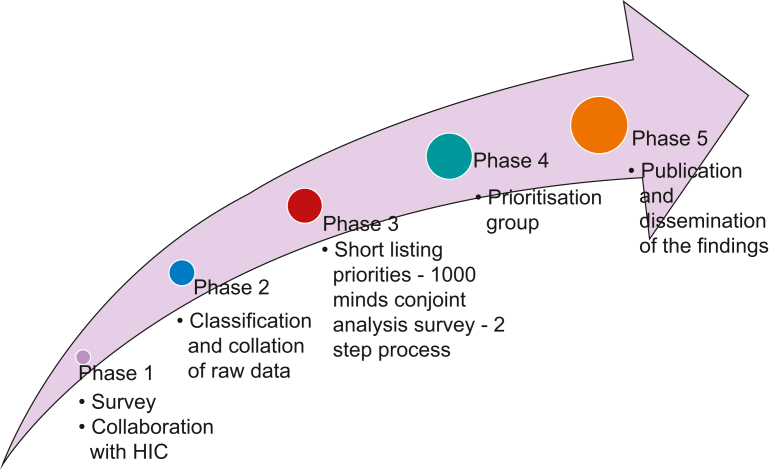
Phases of the perioperative medicine priority gathering partnership in Australia.

HIC, Health Issues Centre.

## Methods

### Study design

A mixed-methods, exploratory-sequential design will be used to analyse qualitative data from open-ended surveys to identify uncertainties/questions relevant to the population in perioperative medical research. Ethical approval to conduct the study was obtained from the Alfred Health (Australia) Human Research Ethics Committee (ID: 171/19).

### Collaboration

Health Issues Centre (HIC) is known as the peak consumer health agency in Victoria, Australia. HIC was developed 40 yr ago, championing consumer and community engagement in healthcare.^34^ A partnership with HIC will enable valuable information and data from various individuals which is imperative to the project, enabling inclusion of minority groups that use health services throughout Australia. As a multiculturally diverse nation, it is essential to make the priorities relatable throughout the country. To do this, we must work with consumers to ensure the survey represents the population. Initially, HIC will work with groups through translation and disseminating the survey at appropriate events and groups. A working group made up of the investigators and representatives of HIC will identify appropriate ways to improve consumer participation for these underrepresented groups in culturally relevant means.

#### Phase 1 (completed)

A survey was designed based on a previous survey used in the JLA PSP in the UK.^25^ The survey was available in an online survey. Study data were collected and managed using Research Electronic Data Capture (REDCap) tools hosted at Monash University.^35^^,^^36^ REDCap is a secure, web-based software platform designed to support data capture for research studies, providing: (1) an intuitive interface for validated data capture; (2) audit trails for tracking data manipulation and export procedures; (3) automated export procedures for seamless data downloads to common statistical packages; and (4) procedures for data integration and interoperability with external sources. The survey was advertised in newsletters, posters, and emails. Healthcare professionals at Alfred Health, Goulburn Health, Australian and New Zealand College of Anaesthetists, and the Royal College of Surgeons all received the survey through an email campaign and college newsletters. The survey was also advertised in presentations to nursing and allied health staff through lectures, advertisements in hospital emails, and department research leads within Australia. Patients and carers were identified through social media campaigns and newsletters by HIC, using targeted social media tactics to address diverse and underrepresented groups. Posters and campaigns were delivered at the Alfred Hospital (metropolitan) and Goulburn Valley Health (rural and regional health service). Patients were also interviewed by research staff at these institutions in preadmission clinics, as inpatients after surgery and at outpatients' appointments. It enabled the researchers to mitigate the responder bias with the survey. This resulted in responses of survey questions from various consumer populations, including researchers, healthcare workers, patients, and carers. All data underwent qualitative data review weekly enabling the identification of underrepresented group responses to further target surveys of underrepresented populations. Collaboration with HIC enabled targeted campaigns to increase responses from underrepresented groups.

#### Phase 2

The data have undergone a framework analysis^37^ approach and alignment to meet the Standardised Endpoints in Perioperative Medicine–Core Outcomes Measures in Perioperative and Anaesthetic Care (StEP–COMPAC) endpoint definitions.^38^, ^39^, ^40^, ^41^, ^42^, ^43^, ^44^, ^45^, ^46^, ^47^ The StEP–COMPAC definitions are expert consensus-based guidelines for clinical outcomes in perioperative medicine.^47^ Each endpoint group has been reviewed and thematically grouped to identify subthemes. Systematic analysis of data identified in the subthemes is currently being undertaken to identify the available evidence in the preceding 6 yr. All unanswered questions will be eligible for phase 3.

### Data collection

#### Phase 1: survey data

An electronic and paper-based survey questionnaire was designed to collate the nominated uncertainties (see Supplementary material, web supplement 1). The survey was publicly available and promoted through hospital platforms and social media campaigns. Participation occurred electronically via the web link, published on posters and social media, or sent as a link via email. For those unable to access the survey as a link, a paper-based survey was made available by contacting one of the authors (SW). Patients and carers were approached when visiting one of two hospitals (Alfred Hospital, Goulburn Valley Health), in the preadmission clinic before surgery, after surgery on the ward, or in the outpatient clinics.

The questions were deliberately open-ended to encourage complete responses about the experience of patients, carers, and clinicians. The survey was derived to encourage people who do not know about research to feel comfortable contributing their ideas and what is important to them. All responses remain confidential and de-identified as answered. A pilot survey study did not occur because a similar survey has been used to conduct JLA in the UK.^26^^,^^27^^,^^48^

We included in the survey additional information about the respondents, such as age group, location, and type of respondent; patient, carer, and health professional, permitting the researchers to monitor the range of respondent types and target publicity towards any underrepresented groups. The analysis of the data did not include identifiable information.

One of the authors (SW) conducted data reviews to enable a review of progress, identify any uncertainty, and clean and categorise the data. Continuous assessment of the responses allowed a targeted approach to underrepresented groups. It assisted in the decision to close the survey early or extend the deadline, depending on the range of responses and the themes identified. The SARS-CoV-2 pandemic halted the project for 12 months, from February 2020 to February 2021. The survey was available from November 2019 to July 2021. Responses were qualitatively reviewed and discussed by the team (HIC, SW, and PM) regularly throughout the survey. It was seen that there were no ‘new’ themes or topics being generated. The pandemic introduced increasing surveys and media coverage that influenced the decrease in the response rate. This led to the researchers ceasing the survey, as the responses were not identifying any new themes and data saturation had been reached. This number, therefore, was felt to be adequate to identify perioperative priorities in this area, as data saturation was evidenced.

Participation in this survey was anonymous and personal data will not be published. Participation in the survey assumed consent and was highlighted in the instructions contained in the survey.

#### Phase 2: analysis of the survey

All questions/uncertainties from the phase 1 survey were imported into NVivo^49^ for analysis. Phase two included authors (SW and PM) conducting a framework analysis^37^ mapping responses into the overarching themes of the StEP–COMPAC endpoints.^38^, ^39^, ^40^, ^41^, ^42^, ^43^, ^44^, ^45^, ^46^, ^47^ All uncertainties are currently undergoing a thematic analysis.^50^ This is to theme the questions of the same objective to identify a single overarching question. All remaining questions will then be explored using literature searches and systematic reviews of the literature in the previous 10 yr. The aim is to identify if any uncertainties have or have not been documented, identified, or answered in the literature. The literature and systematic reviews will be based on worldwide searches for reliable, relevant evidence. Comprehensive analysis and summaries will be made available.

All uncertainties identified in the literature will be removed from the final analysis. All questions remaining, not found in the reviews will be included in a list comprising the remaining 60–90 unanswered questions; these will be eligible for inclusion in phase 3.

#### Phase 3

Phase 3 of the project will adopt a multicriteria decision-making tool: 1000minds software.^51^, ^52^, ^53^ A consensus-based tool for prioritisation will be implemented after validation using a small group who will trial the tool giving individual judgement, consensus weighting, and validation before final implementation. The survey will include all remaining questions, and each question will be weighted on importance by all respondents. A minimum of 100 responses will be required. A minimum of 50% of these must be from patients/carers. After a review of the data, a consensus group with members from HIC and the Australian and New Zealand College of Anaesthetists Clinical Trials Network (ANZCA CTN) (*n*=6) will identify and confirm the top-rated questions—the aim is to identify the most important questions/priorities to take to the final workshop.

#### Phase 4

A face-to-face workshop will involve a meeting facilitated and led by HIC as an independent party, using a nominal group technique to identify and agree on the top 10 perioperative research priorities. The meeting is pencilled to take place in November 2023.

Before the meeting, a working group will work together using Multi-Criteria Decision-Making (MCDM)^54^, ^55^, ^56^, ^57^ in 1000minds to identify the criteria and weights for the meeting (Supplementary Appendix 2).

The MCDM will involve these four components: (1) research questions to be prioritised; (2) criteria (and their levels) by which the research questions are prioritised; (3) weights representing the relative importance of the criteria; (4) decision-makers (‘experts’) and other stakeholders whose preferences are to be represented.

HIC will facilitate a meeting in Melbourne. A core group of researchers, clinicians, and consumers will be identified and invited to attend the meeting to identify the 10 top priorities. An independent moderator will introduce the process using a nominal group technique,^58^ to facilitate the process. The program will be incorporated as per the methods section. The 1000minds software using the previously validated weights and criteria will help the individual work as a team to weigh and discuss the individual questions and work together to review all questions for the survey.

## Results to date

A total of 223 people participated in the survey. Of these, 10 did not complete the questions and supplied comments, one participant did not consent for the responses to be included, and 12 submitted questions relating to paediatrics. A current PSP in paediatrics was published in 2023, and as there were only a handful of questions, paediatrics would not be included.^59^ This leaves 200 participant surveys remaining, totalling 544 narrative lines for review. Of the respondents, 137 were female. Respondent age was from 18 to 79 yr, the average age being 51 yr. Some 60% of all responses were from consumers (patients or carers).

### Data collection

#### Phase 3

After completion of phase 2, the remaining questions/priorities will be made available as a survey. A link to the survey asking individuals to participate will be published in presentations, newsletters, and handouts. A presentation to the clinician-researcher members of the ANZCA CTN will occur at the annual meeting. A survey link will be available in the ANZCA newsletter. Consumers will be identified through HIC, Goulburn Valley Health, and Alfred Health, where an invitation to participate will be published on social media, in newsletters, and posters. Research staff at the two hospitals will also invite patients to complete the survey. Those who need access to the website can contact SW and receive a paper-based survey and a self-addressed return envelope to return the completed form—resulting in a short list of around 30–60 questions.

#### Phase 4—workshop

The HIC will facilitate a meeting in Melbourne. A core group of researchers, clinicians, and consumers will meet to identify the 10 top priorities (Supplementary material, web supplement 2). An invitation to attend the workshop will be advertised in phase 3. All respondents will be asked if they want to participate in the workshop. Healthcare workers will also be invited to attend the meeting. Further consumers will be identified from registries by HIC. All registrants will be collated, and the team will review and identify the most appropriate representative group. Before the meeting, a newsletter explaining the workshop in a concise overview will be made available to the team. The core group will be separated into three groups of four individuals: a medical practitioner, anaesthetist healthcare worker, and two consumers (essentially to empower the consumers). A nominal group technique,^58^ will be used to rank uncertainties for the meeting. The meeting will divide into six workshop stages. Before the meeting, a list of the priorities will be forwarded to all members. (a) The whole group will be welcomed, informed of the workshop process, and designated to one of four smaller groups. (b) Small groups will meet and discuss the priorities, each facilitated by an independent member to encourage participation. The priorities will be reviewed and using the 1000minds software the whole list will be prioritised and scored from 1 to 30. (c) Returning to the large group, the list of priorities will be visually available and merged to identify if there was consensus with any of the priorities. If all groups consecutively listed any of the questions to the lower two-thirds of the list, these priorities will be removed. (d) The group will then review the remaining questions in small groups and prioritise again. Discussions will enable group members to review and discuss the importance or not of the remaining priorities. At the conclusion, the remaining priorities will be agreed and listed. (e) The group will return, and again the priority lists will be reviewed and identified, removing the priorities listed outside the top 10 if consensus. If saturation and agreement of the top 10 priorities are agreed upon before ‘workshop 5’, then the session will conclude at any identified time point. Further rounds and discussions will continue until an agreement between all members has been reached. Details are found in Supplementary material (web supplement 3).

These will be totalled, and the 10 uncertainties with the highest ranking will be identified as the agreed priorities.

### Data analysis plan

All questions/uncertainties from the phase 1 survey were imported into NVivo^49^ for framework analysis^39^^,^^53^ and mapped responses into the overarching themes of the StEP–COMPAC endpoints. Two authors (SW, PM), guided by the current StEP–COMPAC endpoints, independently coded the responses. The authors then completed the framework analysis of all questions individually (phase 2). Data were then reviewed together to identify disagreement, and a meeting with both parties discussed the results and agreed on the coding. If there was any disagreement, an independent observer was available. Each endpoint group is currently under review to thematically^50^ group to identify subthemes and review similar or duplicate questions that will be combined where appropriate.

Systematic literature review summaries will be compiled on all questions. Using an adapted JLA verification form, the primary author (SW) will conduct individualinformal systematic literature review summaries for the preceding 10 yr, completing an individual form for each question. Upon conclusion of the reviews, all questions will meet either no up-to-date, reliable informal systematic reviews of research evidence addressing the uncertainty, or up-to-date informal systematic reviews of research evidence showing that uncertainty exists. Questions with no updated evidence to show that uncertainty exists will be the remaining priorities (*n*=60–90).

**Fig 2:**
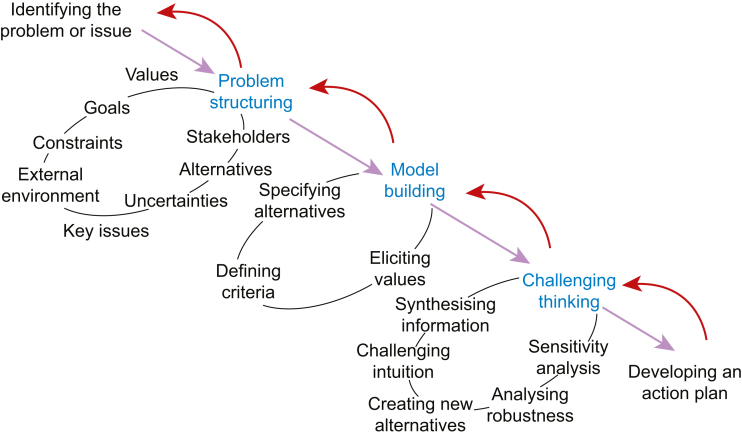
Overview of the MCDA process. MCDA, multi-criteria decision-making.

The remaining priorities will be further analysed by uploading the individual questions into specialised software (1000minds).^51^, ^52^, ^53^ The software will be used to conduct a consensus-based analysis for prioritisation using the multi-criteria decision-making (MCDA) analysis technique (Fig 2).^54^, ^55^, ^56^, ^57^ This will enable the formal structure and solve the decision-making process (phase 3). SW, PM, and HIC agreed to identify four key components (Table 1) of research questions to be prioritised, the criteria and levels to which the criteria are to be prioritised, weights to represent the relative importance of the criteria, and finally, the decision makers whose preferences are to be represented. MCDA uses operational research with its foundations in psychology, mathematics, and economics.^51^, ^52^, ^53^ The software uses the Potentially All Pairwise RanKings of all Possible Alternatives (PAPRIKA) method.^54^ The technique focuses on using weights and associated trade-offs between the criteria, reducing bias from the responders as it is recognised that decisions are made from ‘gut feelings’.^60^ The method uses pairwise comparison or trade-offs to enable the system to judge the preferred priorities and rank the alternatives to estimate weights (‘part-worth utilities’) representing the relative importance of the priorities. Using the 1000minds software, MCDA will be integrated systematically and transparently into the decision-making process. Limitations of the process are the time the survey will take to complete. The highest 30 ranked questions will be available for phase 4.

**Table 1:** Prioritisation criteria.

Relevance	Appropriateness	Significance	Feasibility
1 Low	1 Low	1 Low	1 Low
2 Low-medium	2 Low-medium	2 Low-medium	2 Low-medium
3 Medium	3 Medium	3 Medium	3 Medium
4 Medium-high	4 Medium-high	4 Medium-high	4 Medium-high
5 High	5 High	5 High	5 High

#### Phase 5: dissemination plan

The study findings will be published in a peer-reviewed journal and disseminated to all members of the ANZCA CTN. Public presentations will be held at the ANZCA annual scientific meeting (*n*=1500) ANZCA CTN conference (*n*=200) and public forums at Alfred Health where the talk will be filmed and made available on the Alfred Health and ANZCA CTN websites. Researchers in the perioperative area will use the publication for grant proposals and future research: the relevant local and international conferences, peer-reviewed journals, and clinical guidelines. Findings will be disseminated to funders of research as priorities in this area. These include Australian Clinical Trials Alliance (ACTA), NHMRC, ANZCA, and the Medical Research Future Fund (MRFF). All data will be made available in an appendix to the published work.

## Authors’ contributions

Conceptualisation, methodology, writing the original draft, visualisation: SW

Review and editing, supervision: TB, AF, PM

## Declarations of interest

The authors declare that they have no conflicts of interest.
