# Supplementary material for: A mixed methods study protocol to identify research priorities for perioperative medicine in Australia
Source: BJA Open. 2023 Oct 25;8:100235. doi: 10.1016/j.bjao.2023.100235 (PMC10624594; doi:10.1016/j.bjao.2023.100235)
Supplement: Multimedia component 1 [file mmc1.docx]

**Web supplement 1- Survey**

Help improve care before and after surgery

In Australia the budget for medical research is roughly $1.2 billion a year (NHMRC/MRFF). This funding is to develop and maintain a high standard of excellence in healthcare. Research on the effects of treatments can often overlook the shared interests of patients, carers and clinicians. As a result, questions that they all consider important are not addressed, potentially neglecting many areas of important research.

We would like to collect information from individuals who have had surgery of otherwise care for people undergoing surgery in Australia. This information will be used to guide future research into patient-centred outcomes.

This survey is open to anyone residing in Australia that has an interest in helping to improve Anaesthesia and Perioperative Care. We would like to hear everyone's views whether you are healthcare professional, patient, carer or just an interested member of the community.

The survey will take approximately 10 minutes to complete. We firstly ask some brief details about you (eg. age, postcode, gender & ethnicity). Secondly, we would like you to write down three ideas for future research to improve the well-being of patients having an operation. All information will be gathered and reported anonymously. we ask that you only complete this survey once.

About you
This information will help us identify the range of participants who have responded. All information will be gathered and reported anonymously.

Age (years) Gender

Ethnicity

Postcode
Which of these best describes you?

Male /Female/ Other/ non binary

White/ Black (African)/Asian (Indian, Pakistani, Burmese, other)/Aboriginal/TSI/Other

I know someone who has had surgery
I am a patient who has had surgery
I have or do care for someone who has undergone surgery.
I am a medical professional (Surgeon, Anaesthetist, GP, Nurse ICU, Nurse ward, Nurse Operating theatre, Recovery nurse, Allied health)

Other

(If you are a clinical professional, please tick the box below that is closest to your professional category, or write in "other" if we have admitted your role:)

What area?

If other please describe

Surgeon
Anaesthetist
GP
Nurse ICU
Nurse operating theatre Nurse ward
Recovery nurse
Allied health practitioner

Other

What input are we looking for?
We are looking for ideas and suggestions for future research areas in Anaesthesia and Perioperative Medicine. Our aim is to maximise patient wellbeing from planning an operation, undergoing anaesthesia, waking up following the procedure through to discharge and recovery at home.
Previous surveys have found that the most useful way of collecting this information is for respondents to write short questions describing/outlining what they think should be investigated.

Examples of questions from surveys in other medical areas incude:
1. Are breathing exercises helpful in controlling asthma?
2. What is the evidence that gargling with Aspirin is good for a sore throat? 3. Is it safe to take antidepressants whilst I'm breastfeeding my baby?

Ready to write questions?

Please write a short sentence for each question about going under anaesthetic, and/or care before and after surgery. You may expand on your ideas at the bottom of the page.

1 Question 1 Question 2 Question 3

Consent
By participating in this survey, you are agreeing to allow us to publish your suggestions anonymously, in an online tool for researchers. This will allow researchers to explore the current unanswered questions in their areas of research.
Ethics approval 171/19

I have read the consent section and I am happy to take part
I do not want my suggestions to be included in the online database

**Web supplement 2**

**Workshop 1: Review of the priorities**

All participants will have the priorities distributed within 24 hours of the meeting. Distribution of the priorities allows all members to review and prioritise importance at an individual level. All responses and results will be entered and evaluated using 1000 minds software.

**Workshop 2: small group discussions and ranking**

- In small groups (three groups of up to 4 people – an equal mix of patients, carers and clinicians), each participant, in turn, will contribute their views on the questions they feel are most important for research. Each group will designate a facilitator, who will oversee noting the results. Participants discuss the questions they think are least important in their opinion. Usually, this involves focusing on each participant's top and bottom three questions.
- After this session, each group will rank the list from what they deem to be the most important to the least important for all 30 priorities.

**Workshop 3: whole group review**

- All groups will come together and display each group's results as a list ranked in consecutive order. The results will be aggregated together to produce a combined list.
- The independent meeting chair will present an overview of the results. The whole group is present at this time and can discuss concerns or comments. Groups will then change for all members with an equal balance of patients, carers and clinicians – to discuss and revise the combined ranked list.

**Workshop 4: the second round of small group ranking**

Once again, the new group's members will review the priorities in aggregate order. All members will review and discuss the order and focus discussions on the upper half of the list and prioritise.

**Workshop 5: final whole group review**

- Each group will again submit the results to the system and display the aggregate ranking. The whole team will together discuss the order.
- The result aims to agree on the Top 10
- priorities in rank order by the end of the discussion session. A vote may be used if a consensus cannot be decided upon by discussion.

**Web supplement 3**

Steps to be incorporated prior to the final prioritisation workshop.

1. Participants: Confirming an appropriate cohort of participants. Twenty participants will be invited. Each group must contain a minimum of one of each of Anaesthesiologist, other medical professional and two consumers.

2. A trial MCDM tool

Collate 10-15 research-question ‘vignettes’ (examples) and run a Ranking Survey – a “noise audit” – to capture individual experts’ intuitive rankings. Show them how variable they are! (e.g. see ‘Noisy’ expert judgments) and that, therefore, a better approach is needed!

3. Elicit the prioritisation criteria

As the group discusses the Ranking Survey results. i.e. which research questions should be prioritised or not, and why – start drafting the criteria. Run the same Ranking Survey but this time by consensus to get a pseudo gold standard for step #7 later.

4. Refine the criteria (and their levels)

Refine the criteria and levels – e.g. based on the literature and available evidence and experience. Test the criteria/levels on the above-mentioned vignettes, and refine them as necessary (to improve their validity and reliability). Get the number of levels and the wording right!

5. Individual weightings

Using a preference survey for the group will enable the member to experience making trade-offs between hypothetical research questions rated on the criteria. Enabling a review of areas of agreement and disagreement.

6. Consensus weightings

A decision-making exercise by inviting the members to the 1000minds decision-making tool to weight the criteria and levels by consensus using a survey.

7. Validate the tool

A comparative analysis will be conducted to review the ranking identified in step 3 and the ranking following the incorporation of criteria and weights.

8. Rate research questions

Once there is agreement on the criteria/levels, a Categorization Survey will be run for group to rate all the research questions being considered on the criteria.

9. Average/consensus rating

Resolve disagreements in the ratings of the research questions using the rating-averaging feature in 1000minds or by reaching consensus among the decision-makers.

10. Finalise the priority ranking

Agree on the final ranking of research questions produced by 1000minds applying the weights on the criteria/levels to the average/consensus ratings of the research questions. Perform sensitivity analysis review the steps in diagram 1. Identify if there are any other consideration requiring addressing.
